# Effects of malaria intermittent preventive treatment with dihydroartemisinin-piperaquine on antiretroviral drug concentrations in African pregnant women living with HIV

**DOI:** 10.1128/aac.00337-26

**Published:** 2026-06-12

**Authors:** Linda Stöger, Johannes Mischlinger, Tacilta Nhampossa, Ghyslain Mombo-Ngoma, Meral Esen, André-Marie Tchouatieu, Mercè Brunet, Anete Mendes, Lia Betty Dimessa Mbadinga, Sara Gamberini, Wilfrid Ndzebe Ndoumba, Antía Figueroa-Romero, Bertrand Lell, Heimo Lagler, Rella Zoleko-Manego, Myriam El Gaaloul, Susana Méndez, Núria Botella, Sergi Sanz, Mireia Piqueras, Esperança Sevene, Francisco Saúte, Sebastian G. Wicha, Dolors Soy, Clara Menéndez, Michael Ramharter, Raquel González, on behalf of the MAMAH Study Group

**Affiliations:** 1 ISGlobal 310844, Barcelona, Spain; 2 Facultat de Medicina i Ciències de la Salut, Universitat de Barcelona (UB) 16724, Barcelona, Spain; 3 Centro de Investigación Biomédica en Red de Epidemiología y Salud Pública, Instituto de Salud Carlos III 38176, Madrid, Spain; 4 Center for Tropical Medicine, Bernhard Nocht Institute for Tropical Medicine & I Dept. of Medicine University Medical Center Hamburg-Eppendorf 14888, Hamburg, Germany; 5 German Center for Infection Research, partner site Hamburg-Lübeck-Borstel-Riems, Tübingen, Germany; 6 Centro de Investigação em Saúde de Manhiça (CISM) 339164, Manhiça, Mozambique; 7 Instituto Nacional de Saúde, Ministério de Saúde, Maputo, Mozambique; 8 Centre de Recherches Médicales de Lambaréné (CERMEL) 301568, Lambaréné, Gabon; 9 Research Group Drug Implementation, Section Implementation Research, Bernhard Nocht Institute for Tropical Medicine & I Dept. of Medicine University Medical Center Hamburg-Eppendorf 14888, Hamburg, Germany; 10 Institut für Tropenmedizin, Eberhard Karls University of Tübingen (EKUT) 9188, Tübingen, Germany; 11 Medicines for Malaria Venture (MMV), Geneva, Switzerland; 12 Department of Toxicology and Pharmacology, Hospital Clínic of Barcelona, Barcelona, Spain; 13 Department of Medicine I, Division of Infectious Diseases and Tropical Medicine, Medical University of Vienna 27271, Vienna, Austria; 14 Department of Basic Clinical Practice, Faculty of Medicine, Universitat de Barcelona 16724, Barcelona, Spain; 15 Department of Clinical Pharmacy, Institute of Pharmacy, University of Hamburg 14915, Hamburg, Germany; 16 Division of Medicines, Department of Pharmacy, Pharmacy Service, Hospital Clinic of Barcelona, Universitat de Barcelona 16724, Barcelona, Spain; 17 Servei de Salut Internacional, Hospital Clínic de Barcelona 16493, Barcelona, Spain; The Children's Hospital of Philadelphia, Philadelphia, Pennsylvania, USA

**Keywords:** dihydroartemisinin-piperaquine, antiretroviral therapy, pregnancy, malaria, HIV

## Abstract

Intermittent preventive treatment during pregnancy (IPTp) for malaria prevention with sulfadoxine-pyrimethamine is contraindicated in pregnant women living with HIV receiving cotrimoxazole prophylaxis. A recent clinical trial showed that dihydroartemisinin-piperaquine (DHA-PPQ) for IPTp is a safe and effective option for malaria prevention in this population. The present study evaluated the impact of monthly IPTp with DHA-PPQ on antiretroviral (ARV) drug concentrations in 105 participants recruited in the aforementioned trial in Mozambique and Gabon between January and November 2021. ARV serum concentrations from maternal venous and cord blood collected at the first antenatal care visit and at the end of pregnancy were measured by ultra-high-performance liquid chromatography-tandem mass spectrometry. At the end of pregnancy, median concentrations of dolutegravir were 835.1 ng/mL (248.1–1,687.6) in maternal and 1,204.2 ng/mL (456.4–1,883.3) in cord blood. For lamivudine (3TC), median concentrations were 108.3 ng/mL (28.8–246.1) in maternal and 186.5 ng/mL (81.9–427.6) in cord blood. No significant differences in ARV concentrations were found between the antimalarial treatment arm and the placebo arm. Furthermore, in multivariate exploratory analysis, no independent associations were found between ARV concentrations and participant characteristics. Our study found similar ARV concentrations in both treatment groups, suggesting that DHA-PPQ for monthly IPTp does not affect ARV concentrations in maternal and cord blood in women living with HIV.

**CLINICAL TRIALS:**

This study is registered with ClinicalTrials.gov as NCT03671109.

## INTRODUCTION

Malaria and HIV constitute significant public health challenges in sub-Saharan Africa (SSA), where more than 90% of all malaria cases occur, and 67% of people with HIV live (1, 2). In this region, newly acquired HIV infections are highest in girls and women, accounting for 63% of new HIV infections (3). It is estimated that approximately one million pregnancies are complicated by co-infections of malaria and HIV infection annually in SSA (4, 5). Co-infections during pregnancy pose an increased risk of maternal anemia, placental malaria, adverse pregnancy outcomes such as low birth weight and stillbirth, as well as vertical transmission of HIV (6–9).

Current WHO guidelines recommend three key malaria prevention strategies during pregnancy: intermittent preventive treatment in pregnancy (IPTp) with sulfadoxine-pyrimethamine (SP), the use of insecticide-treated nets, and prompt treatment of infections (10). IPTp-SP is contraindicated in pregnant women living with HIV receiving cotrimoxazole prophylaxis (CTXp) for the prevention of opportunistic infections (11, 12). Since CTXp provides only partial protection against malaria, this population remains without a fully effective malaria prevention option.

In the last decades, alternative drugs for IPTp have been evaluated in women living with HIV. In a multicenter clinical trial conducted from 2009 to 2013 in Kenya, Mozambique, and Tanzania, mefloquine was found to be poorly tolerated and associated with a twofold increased risk of HIV vertical transmission in pregnant women living with HIV on CTXp (13). Concerns about potential drug-drug interactions between antimalarials and antiretrovirals (ARVs) arose; therefore, antiretroviral serum concentrations were assessed in a subgroup of participants, showing decreased nevirapine serum levels in participants in the mefloquine treatment arm at the end of pregnancy (14).

Artemisinin-based combination therapies have previously been shown to be promising candidates for malaria prevention during pregnancy (15, 16). Dihydroartemisinin-piperaquine (DHA-PPQ), administered as monthly IPTp, has recently been found to reduce the risk of malaria infection in two multicenter clinical trials including women with HIV on CTXp (17, 18). Of note, lower DHA concentrations have been reported in pregnant women compared to non-pregnant women (19). Artemisinin derivatives, and particularly PPQ, are metabolized by the same cytochrome P450 enzymes, CYP3A4 and CYP2B6, as NNRTIs such as nevirapine and efavirenz (20, 21). Due to the shared metabolic pathway, co-administration increases the risk of altered drug levels in the body. However, data on pharmacokinetic interactions between DHA-PPQ and newer ARV drugs, such as the integrase strand transfer inhibitor dolutegravir (DTG), during pregnancy were limited at the time the study was designed.

In this study, we evaluated the impact of DHA-PPQ as IPTp on ARV drug levels in a subgroup of pregnant women enrolled in the aforementioned clinical trial conducted in Gabon and Mozambique.

## MATERIALS AND METHODS

### Study sites and participants

This is an observational study nested in a randomized, double-blind, placebo-controlled clinical trial that assessed the safety and efficacy of DHA-PPQ as IPTp in 666 pregnant women living with HIV on daily CTXp, antiretroviral therapy (ART), and using long-lasting insecticide-treated nets in Gabon and Mozambique (MAMAH trial, NCT03671109) (22). At the time of our study, the estimated prevalence of HIV among pregnant women was 29% in Mozambique and 6% in Gabon, whereas *Plasmodium falciparum* infection in women at delivery was 6% in Mozambique and 11% in Gabon (22). The aim of this sub-study was to assess the potential effects of DHA-PPQ on current first-line ARV drugs in pregnant women living with HIV on CTXp. The study includes a subgroup of 105 participants enrolled in the clinical trial from January to November 2021. Pregnant women were included in the study if they were permanent residents in the study area, had a gestational age of up to 28 weeks, HIV seropositive status, and were able and willing to comply with the study procedures.

### Study procedures and ethical considerations

Details of the trial procedures have been described elsewhere (22). Briefly, after signing the informed consent, participants were randomized to receive monthly doses of a 3 day course of DHA-PPQ or placebo as IPTp in addition to daily ART and CTXp. As per national guidelines, first-line efavirenz (EFV)-based ARV treatment regimens consisted of a combination of 600 mg EFV, 300 mg lamivudine (3TC), and 300 mg tenofovir disoproxil fumarate (TDF), administered once daily. First-line DTG-based treatment regimens consisted of 50 mg DTG, 300 mg 3TC, and 300 mg TDF, administered once daily. A zidovudine (AZT)-containing DTG regimen was administered as a 300 mg AZT combined tablet with 150 mg 3TC taken twice daily and a 50 mg DTG tablet taken once daily. ART and CTXp were considered routine medications, and daily drug intake was not supervised in this study. However, treatment adherence reported by the participants was monitored and documented at monthly ANC visits up to the end of the study. For this substudy, 6 mL venous blood samples were collected at recruitment and at the end of pregnancy, as well as 6 mL of cord blood from the newborn. The samples were processed and stored as serum at −80°C until analysis. Data on pregnancy outcome, self-reported treatment adherence, viral load, CD4 cell count, and hemoglobin levels were also collected. Infants testing for HIV by PCR analysis following routine practices were recorded at the age of 6 weeks, 6 months, and 1 year.

### Laboratory procedures

Analysis of ARV and anti-malarial drug levels was performed using an ultra-high-performance liquid chromatography-tandem mass spectrometric method at the Hospital Clinic of Barcelona, Spain. Following sample preparation, chromatography was carried out using an XBridge C18 column. Mass spectrometry analysis was performed using an Acquity Xevo TQ-S Micro system (Waters) in positive ionization mode with multiple reaction monitoring. The lower limits of quantification (LOQ) were 10 ng/mL for DTG, 2.5 ng/mL for 3TC and AZT, 674 ng/mL for EFV, 1 ng/mL for DHA, and 250 ng/mL for PPQ.

### Data management and statistical analysis

The data collection and management for this paper were performed using the OpenClinica open source software (version 3.14, OpenClinica LLC and collaborators, Waltham, MA, USA, https://www.openclinica.com/). Study data were double-entered into the dedicated substudies defined within the MAMAH study database for the corresponding participants. Sociodemographic, clinical, and laboratory characteristics of the participants at enrollment, as well as characteristics and outcomes at the end of pregnancy, were described by treatment arm. Proportions were analyzed using χ² and Fisher’s exact tests, as appropriate. Quantitative variables were displayed as mean or median values and compared using Student’s *t*-test or Wilcoxon rank-sum test, depending on whether they followed a normal distribution or not. Median serum concentrations of ARV drugs were described in all analyzed samples by study arm at recruitment and at the end of pregnancy, applying the Wilcoxon rank-sum test. Serum concentrations below the lower limit of quantification (BQL) were assigned a fixed value of BQL/2 of the analyzed drug. The primary analysis compared median ARV drug serum concentrations (ng/mL) between treatment arms (DHA-PPQ vs placebo) at the end of pregnancy.

Secondary objectives included the comparison of paired data on ARV drug levels and antimalarial drugs by compartment (maternal vs cord blood), using the Wilcoxon signed-rank test. This test was not performed for DHA due to high proportions of undetectable concentrations (≥95%). Additionally, DHA and PPQ median concentrations were described among samples with detectable serum concentrations at the end of pregnancy, applying the Wilcoxon rank-sum test (Table S1). Furthermore, associations of participants’ sociodemographic, clinical, and laboratory characteristics with DTG and 3TC serum levels were explored using logistic regression models. To this end, the outcome variables were defined as having ARV serum concentrations above versus below the study-population medians in maternal and cord blood, respectively. Independent variables with *P*-values <0.25 in the univariate analysis were included in the final logistic regression models. The primary exposure variable, treatment arm (placebo vs DHA-PPQ), was kept in all final models regardless of the univariate *P*-value. Statistical significance was defined by a *P*-value <0.05, and all confidence intervals were calculated at the 95% confidence level. Data analysis was performed using Stata v.16 (Stata Corp., College Station, TX, US).

## RESULTS

**Fig 1:**
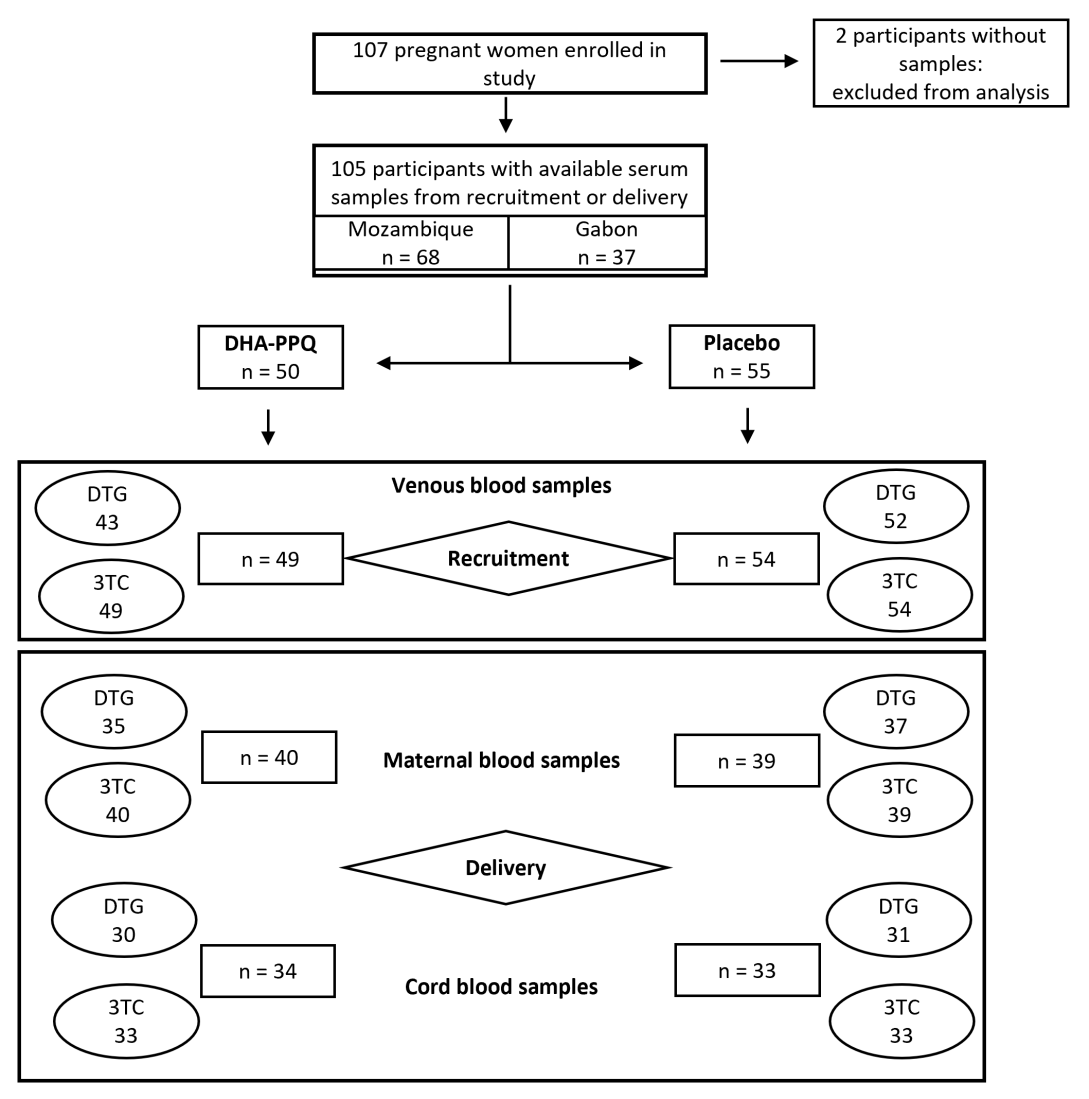
Study profile and overview of analyzed samples. DHA-PPQ, dihydroartemisinin-piperaquine; DTG, dolutegravir; 3TC, lamivudine.

A total of 105 women with available plasma samples at recruitment, the end of pregnancy, or both time points were included in the analysis, 68 from Mozambique and 37 from Gabon (Fig. 1). Participants’ characteristics at baseline are shown in Table 1. The mean age was 29.4 (SD, 5.9) years, with no differences in baseline characteristics between treatment arms. At recruitment, 86 women (81.9%) were already on ART. Ninety-seven participants (92.4%) received a DTG-containing treatment regimen, while eight participants (7.6%) received an EFV-containing treatment regimen. Maternal characteristics and outcomes at the end of pregnancy are shown in Table 2. There were no differences in the proportion of adverse pregnancy outcomes between study arms, and no infant was tested positive for HIV within 12 months after birth.

**TABLE 1:** Baseline sociodemographic and clinical characteristics of study participants^*a*^

	DHA-PPQ (*n* = 50)	Placebo (*n* = 55)	*P*-value
Characteristics at first ANC visit			
Country, *n* (%)			
Gabon	17 (34.0)	20 (36.4)	0.800^*b*^
Mozambique	33 (66.0)	35 (63.6)	
Age (years), mean (SD)	29.3 (6.0)	29.5 (5.9)	0.922^*c*^
Literate (can read and write), *n* (%)	41 (82.0)	46 (83.6)	0.824^*b*^
Gravidity, *n* (%)			
Primigravidae	4 (8.0)	2 (3.6)	0.589*^d^*
1–3 previous pregnancies	30 (60.0)	32 (58.2)	
4 or more previous pregnancies	16 (32.0)	21 (38.2)	
Gestational age (weeks), mean (SD)	18.9 (4.7)	18.5 (4.8)	0.634^*c*^
MUAC (cm), median (IQR)	27.2 (25.2–29.5)	27.0 (24.3–30.1)	0.999^*e*^
BMI, median (IQR)	24.5 (22.4–26.5)	23.9 (21.1–27.6)	0.957^*e*^
Underweight (BMI <18.5), *n* (%)	3 (6.0)	2 (3.6)	0.667^*d*^
Hemoglobin (g/dL), median (IQR)	11.0 (10.1–12.0)	10.8 (9.8–12.1)	0.610^*e*^
Anemia (Hb <11 g/dL), *n* (%)	25 (50.0)	28 (50.9)	0.926^*b*^
Viral load (copies/mL), median (IQR)	0 (0–150)	0 (0–864)	0.654^*e*^
CD4 cell count (cells/µL), mean (SD)	630.3 (260.2)	617.6 (304.4)	0.830^*c*^
On ART at baseline, *n* (%)	42 (84.0)	44 (80.0)	0.595^*b*^
ART regimen			
TDF+3TC+EFV	6 (12.0)	2 (3.6)	0.101^*d*^
TDF+3TC+DTG	43 (86.0)	53 (96.4)	
AZT+3TC+DTG	1 (2.0)	0 (0.0)	

^*a*^ DHA-PPQ, dihydroartemisinin-piperaquine; ANC, antenatal care; MUAC, mid-upper arm circumference; BMI, body mass index; Hb, hemoglobin; ART, antiretroviral therapy; TDF, tenofovir disoproxil fumarate; 3TC, lamivudine; EFV, efavirenz; DTG, dolutegravir; AZT, zidovudine; SD, standard deviation.

^*b*^ *P*-values were calculated using the χ² test according to variable properties and distributions.

^*c*^ *P*-values were calculated using the Student's *t*-test according to variable properties and distributions.

^*d*^ *P*-values were calculated using the Fisher’s exact test according to variable properties and distributions.

^*e*^ *P*-values were calculated using the Wilcoxon rank-sum test according to variable properties and distributions.

**TABLE 2:** Maternal characteristics and outcomes at the end of pregnancy^*d*^

	DHA-PPQ (*n* = 50)	Placebo (*n* = 55)	Overall (*n* = 105)	*P*-value
Characteristics at the end of pregnancy				
Gestational age, median (IQR)	39 (37–41)	39 (38–40)	39 (37–41)	0.613^*a*^
Hemoglobin (g/dL), median (IQR)	11.0 (10.0–11.6)	10.9 (10.2–11.5)	10.9 (10.1–11.6)	0.479^*a*^
Anemia (Hb <11 g/dL), *n* (%)	23 (50)	25 (51.0)	48 (50.5)	0.921^*b*^
Viral load (copies/mL), median (IQR)	0 (0–0)	0 (0–0)	0 (0–0)	0.905^*a*^
CD4 cell count (cells/µL), median (IQR)	487 (337–819)	580 (375–721)	543 (343–797)	0.290^*a*^
Adherence to ART, *n* (%)				0.829^*a*^
Above 80%	41 (82.0)	44 (80.0)	85 (81.0)	
Below 80%	4 (8.0)	5 (9.1)	9 (8.6)	
No data	5 (10.0)	6 (10.9)	11 (10.5)	
Adherence to CTX, *n* (%)				
Above 80%	36 (72.0)	42 (76.4)	78 (74.3)	0.461^*a*^
Below 80%	9 (18.0)	7 (12.7)	16 (15.2)	
No data	5 (10.0)	6 (10.9)	11 (10.5)	
Pregnancy outcomes, *n* (%)				0.899^*c*^
Live births	45 (90.0)	48 (87.2)	93 (88.6)	
Preterm	1 (2.0)	0 (0.0)	1 (1.0)	
Stillbirths	1 (2.0)	2 (3.6)	3 (2.9)	
Spontaneous abortions	1 (2.0)	1 (1.8)	2 (1.9)	
Miscarriages	1 (2.0)	0 (0.0)	1 (1.0)	
No data	2 (4.0)	4 (7.3)	6 (5.7)	
HIV vertical transmission				
Infant HIV PCR positive (within 12 months), *n* (%)	0 (0.0)	0 (0.0)	0 (0.0)	

^*a*^ *P*-values were calculated using the Wilcoxon rank-sum test according to variable properties and distributions.

^*b*^ *P*-values were calculated using the χ² test according to variable properties and distributions.

^*c*^ *P*-values were calculated using the Fisher’s exact test according to variable properties and distributions.

^*d*^ DHA-PPQ, dihydroartemisinin-piperaquine; Hb, hemoglobin; ART, antiretroviral therapy; CTX, cotrimoxazole.

### ARV drug concentrations at enrollment

Serum samples collected at recruitment were available from 103 women and analyzed for DTG (*n* = 95) and 3TC (*n* = 103) concentrations. Twenty-four (25.3%) samples had DTG concentrations, and 26 (25.2%) samples had 3TC concentrations below the lower limit of quantification. Overall median serum concentrations were 775.8 ng/mL (IQR, 5.0–1,918.2) for DTG and 51.3 ng/mL (IQR, 1.3–401.9) for 3TC, with no difference between study arms (Table 3).

**TABLE 3:** Antiretroviral drug concentrations (ng/mL) at recruitment^*a*^^,^^*b*^

Recruitment - Maternal blood			
	DHA-PPQ	Placebo	*P*-value
DTG	n = 43	n = 52	
Serum concentrations, median (IQR)	875.8 (5.0–2,221.9)	641.4 (39.7–1,525.4)	0.456
Proportion undetectable, *n* (%), n	11 (25.6)	13 (25.0)	
3TC	n = 49	n = 54	
Serum concentrations, median (IQR)	62.3 (8.5–431.0)	49.7 (1.3–262.6)	0.697
Proportion undetectable, *n* (%)	12 (24.5)	14 (25.9)	

^*a*^ Antiretroviral serum concentrations are shown as median (IQR). Comparisons between treatment arms were performed using the Wilcoxon rank-sum test.

^*b*^ DHA-PPQ, dihydroartemisinin-piperaquine; DTG, dolutegravir; 3TC, lamivudine.

### ARV drug concentrations at the end of pregnancy

Regarding samples collected at the end of pregnancy, 11 (15.3%) out of 72 maternal serum samples and 6 (9.8%) out of 61 cord serum samples that were analyzed for DTG were below the lower limit of quantification. For 3TC, 8 (10.1%) out of 79 analyzed maternal serum samples and 5 (7.6%) out of 66 cord serum samples were below the lower limit of quantification. At the end of pregnancy, overall median DTG serum concentrations in maternal blood were 835.1 ng/mL (248.1–1,687.6), and in cord blood 1,204.2 ng/mL (456.4–1,883.3). Median 3TC serum concentration in maternal blood was 108.3 ng/mL (28.8–246.1), and in cord blood 186.5 ng/mL (81.9–427.6). No statistically significant differences were found between treatment arms (Table 4).

**TABLE 4:** Antiretroviral drug concentrations (ng/mL) at the end of pregnancy^*a*^^,^^*b*^

End of pregnancy - Maternal blood			
	DHA-PPQ	Placebo	*P*-value
DTG	n = 35	n = 37	
Serum concentrations, median (IQR)	784.8 (182.4–1,995.0)	862.2 (362.2–1,584.3)	0.753
Proportion undetectable, *n* (%)	7 (20.0)	4 (10.8)	0.279
3TC	n = 40	n = 39	*P*-value
Median serum concentrations, (IQR)	75.4 (17.4–241.2)	128.6 (49.2–246.1)	0.505
Proportion undetectable, (%)	5 (12.5)	3 (7.7)	0.712

End of pregnancy - Cord blood			
	DHA-PPQ	Placebo	*P*-value
DTG	n = 30	n = 31	
Serum concentrations, median (IQR)	1,166.1 (421.1–1,895.2)	1,260.5 (544.6–1,832.1)	0.940
Proportion undetectable, *n* (%)	3 (10.0)	3 (9.7)	1.000
3TC	n = 33	n = 33	*P*-value
Serum concentrations, median (IQR)	231.5 (81.9–431.4)	163.9 (86.9–416.9)	0.839
Proportion undetectable, *n* (%)	2 (6.1)	3 (9.1)	1.000

^*a*^ Antiretroviral serum concentrations in maternal and cord blood are shown as median (IQR), and undetectable proportions are shown as *n* (%). Comparisons between treatment arms were performed using the Wilcoxon rank-sum test for continuous variables and Fisher’s exact or χ² tests for proportions.

^*b*^ DHA-PPQ, dihydroartemisinin-piperaquine; DTG, dolutegravir; 3TC, lamivudine.

AZT and EFV were below the lower limit of quantification in all analyzed samples.

Comparing ARV serum concentrations between compartments in paired samples revealed overall higher ARV serum concentrations in cord blood compared to maternal blood for DTG (1,204.2 ng/mL [456.4–1,883.3] vs 859.8 ng/mL [299.5–1,707.4], *P* < 0.001) and for 3TC (186.5 ng/mL [81.9–427.6] vs 127.4 ng/mL [35.5–246.1], *P* < 0.001) (Table 5).

**TABLE 5:** DTG, 3TC, and PPQ concentrations (ng/mL) at the end of pregnancy by compartment (paired data)^*a*^^,^^*b*^

	Maternal serum	Cord serum	*P*-value
	Median (IQR)	Median (IQR)	
DTG serum concentration, (*n* = 61)	859.8 (299.5–1,707.4)	1,204.2 (456.4–1,883.3)	<0.001
3TC serum concentration, (*n* = 66)	127.4 (35.5–246.1)	186.5 (81.9–427.6)	<0.001
PPQ serum concentration, (*n* = 34)	43.3 (15.5–60.3)	13.1 (6.5–36.1)	0.005

^*a*^ Antiretroviral serum concentrations are shown as median (IQR). Comparisons between maternal and cord blood compartments were performed using the Wilcoxon signed-rank test.

^*b*^ DTG, dolutegravir; 3TC, lamivudine; PPQ, piperaquine.

### DHA and PPQ drug concentrations

When analyzing the antimalarial drugs DHA and PPQ in the intervention arm, only two maternal and one cord blood sample had quantifiable serum concentrations of the short half-life drug DHA. Thirty-nine (97.5%) and 33 (97.0%) samples had quantifiable serum concentrations of PPQ in maternal and cord blood, respectively (Table S1).

In a paired sample analysis, PPQ concentrations were higher in maternal compared to cord blood (43.3 ng/mL [15.5–60.3] vs 13.1 [6.5–36.1], *P* = 0.005) (Table 5).

### Determinants of ARV drug levels

Assessment of factors associated with DTG and 3TC serum concentrations above versus below the study population medians using exploratory logistic regression is shown in the Table S2a through d. In univariate analyses, weak associations were observed for anemia and days since last IPTp. However, none of the covariates remained associated with the outcome in the final multivariate models.

## DISCUSSION

This is, to our knowledge, the first study evaluating antiretroviral drug levels at the end of pregnancy in African women living with HIV taking DHA-PPQ as IPTp together with daily cotrimoxazole prophylaxis. DHA-PPQ did not seem to affect ARV serum concentrations since no differences were found between treatment arms. We found higher ARV concentrations but lower PPQ concentrations in cord serum compared to maternal serum.

No differences were found in the serum concentrations of both DTG and 3TC between DHA-PPQ and placebo arms. Most importantly, there were no cases of vertical transmission among the participants included in this study. Furthermore, pregnancy outcomes in our cohort were similar between treatment arms. These findings confirm the suitability of DHA-PPQ for IPTp in combination with daily CTXp and ART in pregnant women living with HIV, which in two recent clinical trials conducted in Gabon, Mozambique, Malawi, and Kenya was found to be safe and to reduce overall malaria risk in this vulnerable population (17, 18). Our results are in contrast with previous findings for the antimalarial drug mefloquine, which was assessed for IPTp and was shown to reduce nevirapine serum concentrations (14).

While our study did not find changes in ARV serum concentrations between treatment arms under conditions of unsupervised antiretroviral drug intake, a drug-drug interaction study evaluating the effect of DHA-PPQ on DTG exposure among pregnant women living with HIV in Malawi found a modest increase in DTG exposure when co-administered with DHA-PPQ, with no clinically significant adverse events (16).

Both DTG and 3TC showed higher median serum concentrations in cord blood compared to maternal blood. This may be explained by high ARV drug transfer through the placenta and was shown in a previous study where DTG concentrations at delivery were 1.25-fold higher in cord blood compared to maternal blood, and prolonged elimination was observed in infant blood, possibly due to lower drug-metabolizing enzyme activity in the newborn (23, 24). Elevated DTG exposure in cord blood may have implications for potential toxicities and calls for further investigation into possible safety concerns. PPQ, in contrast, was detected at higher levels in maternal compared to cord plasma. While there are no reports on PPQ concentrations in cord blood, the reasons for the observed differences may be related to protein-binding characteristics that disfavor transfer through the placenta. PPQ is highly protein-bound, and protein binding has been described to influence drug transfer through the placental tissue, which may explain decreased concentrations in cord blood compared to maternal blood (25, 26).

We performed an exploratory analysis to identify participant characteristics independently associated with altered ARV serum concentrations. While in the multivariate analysis no statistically significant associations were observed, two factors showed a trend toward altered ARV concentrations in the univariate analysis. Anemia showed a tendency toward lower ARV concentrations, possibly reflecting overall suboptimal adherence to drugs taken during pregnancy, such as iron supplements. The number of days since the last monthly IPTp administered showed a trend toward higher 3TC concentrations, in the opposite direction than expected, albeit not statistically significant.

Given the relatively small number of participants, these exploratory results should be interpreted with caution. Future studies with larger sample sizes could further explore potential associations and increase statistical power.

The results of our study strengthen the evidence for the safety of DHA-PPQ without affecting overall serum concentrations of ARV drugs.

Our study has some limitations. A formal pharmacokinetic analysis using a rich-sampling protocol as originally planned could not be conducted due to recruitment challenges and the resulting small sample size achieved. The main reason was the low acceptability of repeated blood sampling among healthy women as the complex sampling schedule would have required hospitalization for the days of sampling. Furthermore, the start of recruitment had to be considerably delayed due to the COVID-19 pandemic, resulting in a shorter recruitment period and a smaller sample size than planned. Importantly, our data on ARV drug concentrations were obtained under real-world conditions without information on the timing of ARV drug intake. Consequently, we could not adjust for time of drug intake as a confounder in relation to blood draw or the ARV concentration itself in this analysis. Nevertheless, our findings consistently showed no significant effect of antimalarial treatment on ARV serum levels both in the univariate continuous analysis and the adjusted multivariate logistic regression.

Overall, our study contributes important information on the safety of co-administration of DHA-PPQ to daily CTX in pregnant women receiving antiretroviral therapy. The findings suggest that DHA-PPQ is a safe option for IPTp in women living with HIV in sub-Saharan Africa.

## Data Availability

De-identified individual data and the data dictionary will be made available to others upon reasonable request to the corresponding author. A data transfer agreement will be signed between the MAMAH consortium and the requesting institution before data sharing.

## SUPPLEMENTAL MATERIAL

Supplemental material aac.00337-26-s0001.docx Tables S1 and S2.

ASM does not own the copyrights to Supplemental Material that may be linked to, or accessed through, an article. The authors have granted ASM a non-exclusive, world-wide license to publish the Supplemental Material files. Please contact the corresponding author directly for reuse.

## References

[1] WHO. 2023. World malaria report 2023. World Health Organization

[2] Oladipo HJ, Tajudeen YA, Oladunjoye IO, Yusuff SI, Yusuf RO, Oluwaseyi EM, AbdulBasit MO, Adebisi YA, El-Sherbini MS. 2022. Increasing challenges of malaria control in sub-Saharan Africa: Priorities for public health research and policymakers. Ann Med Surg (Lond) 81:104366. doi:10.1016/j.amsu.2022.104366 36046715 PMC9421173

[3] UNAIDS. 2023. The path that ends AIDS. UNAIDS Global AIDS update

[4] Kwenti TE. 2018. Malaria and HIV coinfection in sub-Saharan Africa: prevalence, impact, and treatment strategies. Res Rep Trop Med 9:123–136. doi:10.2147/RRTM.S154501 30100779 PMC6067790

[5] Uneke CJ, Ogbonna A. 2009. Malaria and HIV co-infection in pregnancy in sub-Saharan Africa: impact of treatment using antimalarial and antiretroviral agents. Trans R Soc Trop Med Hyg 103:761–767. doi:10.1016/j.trstmh.2008.06.017 18707747

[6] Ssentongo P, Ba DM, Ssentongo AE, Ericson JE, Wang M, Liao D, Chinchilli VM. 2020. Associations of malaria, HIV, and coinfection, with anemia in pregnancy in sub-Saharan Africa: a population-based cross-sectional study. BMC Pregnancy Childbirth 20:379. doi:10.1186/s12884-020-03064-x 32600355 PMC7324981

[7] Brentlinger PE, Behrens CB, Micek MA. 2006. Challenges in the concurrent management of malaria and HIV in pregnancy in sub-Saharan Africa. Lancet Infect Dis 6:100–111. doi:10.1016/S1473-3099(06)70383-8 16439330

[8] Nkhoma ET, Kalilani-Phiri L, Mwapasa V, Rogerson SJ, Meshnick SR. 2012. Effect of HIV infection and Plasmodium falciparum parasitemia on pregnancy outcomes in Malawi. Am J Trop Med Hyg 87:29–34. doi:10.4269/ajtmh.2012.11-0380 22764288 PMC3391053

[9] Mbachu II, Ejikunle SD, Anolue F, Mbachu CN, Dike E, Ejikem E, Okeudo C. 2020. Relationship between placenta malaria and mother to child transmission of HIV infection in pregnant women in South East Nigeria. Malar J 19:1–8. doi:10.1186/s12936-020-03171-2 32103782 PMC7045610

[10] WHO. 2022. Updated WHO recommendations for malaria chemoprevention among children and pregnant women

[11] WHO. 2013. WHO policy brief for the implementation of intermittent preventive treatment of malaria in pregnancy using sulfadoxine-pyrimethamine (IPTp-SP)

[12] González R, Sevene E, Jagoe G, Slutsker L, Menéndez C. 2016. A public health paradox: the women most vulnerable to malaria are the least protected. PLoS Med 13:e1002014. doi:10.1371/journal.pmed.1002014 27139032 PMC4854455

[13] González R, Desai M, Macete E, Ouma P, Kakolwa MA, Abdulla S, Aponte JJ, Bulo H, Kabanywanyi AM, Katana A, Maculuve S, Mayor A, Nhacolo A, Otieno K, Pahlavan G, Rupérez M, Sevene E, Slutsker L, Vala A, Williamsom J, Menéndez C. 2014 Intermittent preventive treatment of malaria in pregnancy with mefloquine in HIV-infected women receiving cotrimoxazole prophylaxis: a multicenter randomized placebo-controlled trial. PLoS Med 11:e1001735. doi:10.1371/journal.pmed.1001735 25247995 PMC4172537

[14] Haaland RE, Otieno K, Martin A, Katana A, Dinh C, Slutsker L, Menendez C, Gonzalez R, Williamson J, Heneine W, et al.. 2018. Short communication: reduced nevirapine concentrations among HIV-positive women receiving mefloquine for intermittent preventive treatment for malaria control during pregnancy. AIDS Res Hum Retroviruses 34:912–915. doi:10.1089/AID.2018.0042 30173559 PMC6238614

[15] Kakuru A, Jagannathan P, Muhindo MK, Natureeba P, Awori P, Nakalembe M, Opira B, Olwoch P, Ategeka J, Nayebare P, et al.. 2016. Dihydroartemisinin-piperaquine for the prevention of malaria in pregnancy. N Engl J Med 374:928–939. doi:10.1056/NEJMoa1509150 26962728 PMC4847718

[16] Banda CG, Nkosi D, Allen E, Workman L, Madanitsa M, Chirwa M, Kapulula M, Muyaya S, Munharo S, Wiesner L, et al.. 2022. Effect of dihydroartemisinin/piperaquine for malaria intermittent preventive treatment on dolutegravir exposure in pregnant women living with HIV. J Antimicrob Chemother 77:1733–1737. doi:10.1093/jac/dkac081 35288747 PMC9155593

[17] González R, Nhampossa T, Mombo-Ngoma G, Mischlinger J, Esen M, Tchouatieu A-M, Mendes A, Figueroa-Romero A, Zoleko-Manego R, Lell B, et al.. 2024. Safety and efficacy of dihydroartemisinin-piperaquine for intermittent preventive treatment of malaria in pregnant women with HIV from Gabon and Mozambique: a randomised, double-blind, placebo-controlled trial. Lancet Infect Dis 24:476–487. doi:10.1016/S1473-3099(23)00738-7 38224706

[18] Barsosio HC, Madanitsa M, Ondieki ED, Dodd J, Onyango ED, Otieno K, Wang D, Hill J, Mwapasa V, Phiri KS, et al.. 2024. Chemoprevention for malaria with monthly intermittent preventive treatment with dihydroartemisinin–piperaquine in pregnant women living with HIV on daily co-trimoxazole in Kenya and Malawi: a randomised, double-blind, placebo-controlled trial. The Lancet 403:365–378. doi:10.1016/S0140-6736(23)02631-4 PMC10865779 38224710

[19] Tarning J, Rijken MJ, McGready R, Phyo AP, Hanpithakpong W, Day NPJ, White NJ, Nosten F, Lindegardh N. 2012. Population pharmacokinetics of dihydroartemisinin and piperaquine in pregnant and nonpregnant women with uncomplicated malaria. Antimicrob Agents Chemother 56:1997–2007. doi:10.1128/AAC.05756-11 22252822 PMC3318332

[20] Lee TMN, Huang L, Johnson MK, Lizak P, Kroetz D, Aweeka F, Parikh S. 2012. In vitro metabolism of piperaquine is primarily mediated by CYP3A4. Xenobiotica 42:1088–1095. doi:10.3109/00498254.2012.693972 22671777 PMC5087332

[21] Byakika-Kibwika P, Lamorde M, Mayito J, Nabukeera L, Namakula R, Mayanja-Kizza H, Katabira E, Ntale M, Pakker N, Ryan M, et al.. 2012. Significant pharmacokinetic interactions between artemether/lumefantrine and efavirenz or nevirapine in HIV-infected Ugandan adults. J Antimicrob Chemother 67:2213–2221. doi:10.1093/jac/dks207 22687893 PMC3465101

[22] González R, Nhampossa T, Mombo-Ngoma G, Mischlinger J, Esen M, Tchouatieu AM, Pons-Duran C, Dimessa LB, Lell B, Lagler H, et al.. 2021. Evaluation of the safety and efficacy of dihydroartemisinin-piperaquine for intermittent preventive treatment of malaria in HIV-infected pregnant women: protocol of a multicentre, two-arm, randomised, placebo-controlled, superiority clinical trial (MAMAH project). BMJ Open 11:e053197. doi:10.1136/bmjopen-2021-053197 PMC8611429 34815285

[23] Mulligan N, Best BM, Wang J, Capparelli EV, Stek A, Barr E, Buschur SL, Acosta EP, Smith E, Chakhtoura N, et al.. 2018. Dolutegravir pharmacokinetics in pregnant and postpartum women living with HIV. AIDS 32:729–737. doi:10.1097/QAD.0000000000001755 29369162 PMC5854536

[24] Ikumi NM, Anumba D, Matjila M. 2022. Pharmacokinetics and placental transfer of dolutegravir in pregnancy. J Antimicrob Chemother 77:283–289. doi:10.1093/jac/dkab365 34618029

[25] Hoglund RM, Adam I, Hanpithakpong W, Ashton M, Lindegardh N, Day NPJ, White NJ, Nosten F, Tarning J. 2012. A population pharmacokinetic model of piperaquine in pregnant and non-pregnant women with uncomplicated Plasmodium falciparum malaria in Sudan. Malar J 11:398. doi:10.1186/1475-2875-11-398 23190801 PMC3551687

[26] Garland M. 1998. Pharmacology of drug transfer across the placenta. Obstet Gynecol Clin North Am 25:21–42. doi:10.1016/s0889-8545(05)70356-9 9547758

