# Supplementary material for: Effects of malaria intermittent preventive treatment with dihydroartemisinin-piperaquine on antiretroviral drug concentrations in African pregnant women living with HIV
Source: Antimicrob Agents Chemother. 2026 Jun 12;70(7):e00337-26. doi: 10.1128/aac.00337-26 (PMC13321826; doi:10.1128/aac.00337-26)
Supplement: Supplemental material — Tables S1 and S2. [file aac.00337-26-s0001.docx]

**Supplemental Material**

|  | **Venous blood**  (n=2) | **Cord blood**  (n=1) |
| --- | --- | --- |
| ***DHA*** | 11.4 (11.0 - 11.8) | 11.2 (11.2 - 11.2) |
| Proportion undetectable, n (%) | 38 (95) | 38 (97.4) |
|  |  |  |
|  | **Venous blood**  (n=39) | **Cord blood**  (n=33) |
| ***PPQ*** | 36.4 (14.4 - 59.9) | 13.5 (7.9 - 36.1) |
| Proportion undetectable, n (%) | 1 (2.5) | 1 (2.9) |

**Table S1.** Serum concentrations (ng/mL) of DHA and PPQ in maternal and cord blood at the end of pregnancy in the treatment arm.
Detectable DHA and PPQ serum concentrations are shown as median [IQR].

Abbreviations: DHA, dihydroartemisinin; PPQ, piperaquine

**Table S2. Factors associated with altered antiretroviral drug serum concentrations (above versus below median)**

1. ***DTG maternal blood (median conc. 835.1 ng/mL)***

| **DTG MATERNAL BLOOD** | | | | |  |
| --- | --- | --- | --- | --- | --- |
| **Characteristic** | **Below median**  **(n=36)** | **Above median**  **(n=36)** | ***P*-value** | **Univariate OR (95% CI)** | **Adjusted OR (95% CI)** |
|  |  |  |  |  |  |
| **Treatment (DHA-PPQ)**, n (%) |  |  |  |  |  |
| **Placebo** | 17 (47.2) | 20 (55.6) | 0.479*^a^* | Reference | Reference |
| **DHA-PPQ** | 19 (52.8) | 16 (44.4) |  | 0.72 (0.28-1.81) | 0.67 (0.24- 1.90) |
| **Country (Gabon)**, n (%) |  |  |  |  |  |
| **Mozambique** | 27 (75.0) | 25 (69.4) | 0.599*^a^* | Reference | - |
| **Gabon** | 9 (25.0) | 11 (30. 6) |  | 1.32 (0.47-3.72) | - |
| **Age (years)**, mean (SD) | 28 (6.0) | 29.94 (6.6) | 0.194*^b^* | 1.05 (0.97-1.13) | 1.02 (0.94- 1.11) |
| **BMI**, median [IQR] | 24.3 [22.1-25.8] | 4.9 [20.0-27.6] | 0.542*^c^* | 1.05 (0.95-1.16) | - |
| **MUAC**, median [IQR] | 26.3 [25.1-28.4] | 27.1 [25.0-30.7] | 0.503*^c^* | 1.07 (0.94-1.21) | - |
| **Gestational age recruitment**, mean (SD) | 18.5 (4.0) | 19.3 (4.7) | 0.453*^b^* | 1.04 (0.94-1.16) | - |
| **Gestational age delivery**, median [IQR] | 39 [37.5-4] | 39.0 [37.5-41.0] | 0.863*^c^* | 0.95 (0.83-1.10) | - |
| **Number of previous gestations**, median [IQR] | 2.5 [1.5-3] | 3 [1.5-4] | 0.450*^c^* | 1.07 (0.84-1.37) | - |
| **Literacy status (Literate)**, n (%) |  |  |  |  | - |
| **cannot read/write** | 8 (6.5) | 5 (6.5) | 0.358*^a^* | 1.77 (0.52-6.05) |  |
| **can read/write** | 28 (29.5) | 31 (29.5) |  |  |  |
| **Anemia (Hb<11 g/dL) at delivery**, n (%) |  |  |  |  |  |
| **not anemic** | 12 (33.3) | 20 (55.6) | 0.058*^a^* | 0.40 (0.15-1.04) | 0.39 (0.14-1.08) |
| **anemic** | 24 (66.7) | 16 (44.4) |  |  |  |
| **HIV viral load at delivery**, median [IQR] | 0 [0-0] | 0 [0-0] | 0.283*^c^* | 1.00 (0.9998-1.0003) | - |
| **CD4 cells count at delivery**, median [IQR] | 590 [401-977] | 490 [337-706] | 0.291*^c^* | 0.9990 (0.9976-1.0003) | 0.9990 (0.9975- 1.0004) |
| **Adherence to ART**, n (%) |  |  |  |  | - |
| **above 80%** | 32 (91.4) | 31 (93.9) | 0.528*^d^* | 1.45 (0.23-9.30) | - |
| **below 80%** | 3 (8.6) | 2 (6.1) |  | Reference | - |
| **Days since last IPTp dose**, median [IQR] | 24 [14-33] | 25 [17-31] | 0.896*^c^* | 1.01 (0.99-1.03) | - |

1. ***DTG cord blood (median conc. 1204.2 ng/mL)***

| **DTG CORD BLOOD** | | | | | |
| --- | --- | --- | --- | --- | --- |
| **Characteristic** | **Below median**  **(n=31)** | **Above median**  **(n=30)** | ***P*-value** | **Univariate OR (95% CI)** | **Adjusted OR (95% CI)** |
| **Treatment (DHA-PPQ)**, n (%) |  |  |  |  |  |
| **Placebo** | 15 (48.4) | 16 (53.3) | 0.699*^a^* | Reference | Reference |
| **DHA-PPQ** | 16 (51.6) | 14 (46.7) |  | 0.82 (0.30-2.24) | 0.84 (0.28 2.54) |
| **Country (Gabon)**, n (%) |  |  |  |  |  |
| **Mozambique** | 24 (77.4) | 23 (76.7) | 0.944*^a^* | Reference | - |
| **Gabon** | 7 (22.6) | 7 (23.3) |  | 1.04 (0.32-3.44) | - |
| **Age**, mean (SD) | 28.2 (6.8) | 29.6 (6.0) | 0.373*^b^* | 1.04 (0.96-1.12) | - |
| **BMI**, median [IQR] | 24.4 [21.9- 26.7] | 24.5 [22.0-26.5] | 0.858*^c^* | 0.97 (0.85-1.11) | - |
| **MUAC**, median [IQR] | 27.8 (3.5) | 27.4 (3.4) | 0.658*^c^* | 0.97 (0.83-1.12) | - |
| **Gestational age recruitment**, mean (SD) | 18.9 (4.4) | 18.9 (4.4) | 0.578*^b^* | 1.03 (0.92-1.16) | - |
| **Gestational age delivery**, median [IQR] | 40 [38-41] | 39 [37-40] | 0.166*^c^* | 0.85 (0.69-1.06) | .81 (.28-2.54) |
| **Number of previous gestations**, median [IQR] | 2 [1-3] | 3 [2-4] | 0.249*^c^* | 1.08 (0.83-1.42) | - |
| **Literacy status (Literate)**, n (%) |  |  |  |  |  |
| **cannot read/write** | 6 (19.4) | 4 (13.3) | 0.525*^a^* | Reference | - |
| **can read/write** | 25 (80.65) | 26 (86.67) |  | 1.56 (0.39-6.20) | - |
| **Anemia (Hb<11 g/dL) at delivery**, n (%) |  |  |  |  |  |
| **not anemic** | 11 (35.48) | 16 (53.33) | 0.161*^a^* | Reference | Reference |
| **anemic** | 20 (64.52) | 14 (46.67) |  | 0.48 (0.17-1.34) | .36 (.11-1.14) |
| **HIV viral load at delivery**, median [IQR] | 0 [0-0] | 0 [0-0] | 0.663*^c^* | 1.0001 (0.9998-1.0004) | - |
| **CD4 cells count at delivery**, median [IQR] | 543 [397-706] | 473 [329-797] | 0.717*^c^* | 0.9995 (0.9981-1.0009) | - |
| **Adherence to ART,** n (%) |  |  |  |  |  |
| **above 80%** | 26 (86.67) | 27 (96.43) | 0.354*^d^* | 4.15 (0.43-39.67) | 7.41 (.67-81.41) |
| **below 80%** | 4 (13.33) | 1 (3.57) |  | Reference | Reference |
| **Days since last IPTp dose**, median [IQR] | 24 [15-32] | 24 [13-32] | 0.794*^c^* | 1.01 (0.99-1.03) | - |

1. ***3TC maternal blood (median conc. 108.3 ng/mL)***

| **3TC MATERNAL BLOOD** | | | | | |
| --- | --- | --- | --- | --- | --- |
| **Characteristic** | **Below median**  **(n=39)** | **Above median**  **(n=40)** | ***P*-value** | **Univariate OR (95% CI)** | **Adjusted OR (95% CI)** |
| **Treatment (DHA-PPQ)**, n (%) |  |  |  |  |  |
| **Placebo** | 18 (45.0) | 21 (53.9) | 0.432*^a^* | Reference | Reference |
| **DHA-PPQ** | 22 (55.0) | 18 (46.2) |  | 0.70 (0.29-1.70) | 1.17 (.41-3.29) |
| **Country (Gabon)**, n (%) |  |  |  |  |  |
| **Mozambique** | 31 (77.5) | 26 (66.7) | 0.283*^a^* | Reference | - |
| **Gabon** | 9 (22.5) | 13 (33.3) |  | 1.72 (0.64-4.67) | - |
| **Age**, mean (SD) | 29.1 (6.7) | 29.3 (5.7) | 0.882*^b^* | 1.01 (0.94-1.08) | - |
| **BMI**, median [IQR] | 24.5 [22.1-26.1] | 24.3 [21.2- 27.5] | 0.973*^c^* | 1.02 (0.93-1.12) | - |
| **MUAC**, mean (SD) | 27.5 (3.4) | 27.6 (3.9) | 0.889*^b^* | 1.01 (0.89-1.14) | - |
| **Gestational age recruitment**, mean (SD) | 19 (4.2) | 18.6 (4.6) | 0.661*^b^* | 0.98 (0.88-1.08) | - |
| **Gestational age delivery**, median [IQR] | 39 [37.5-41] | 39 [37-41] | 0.621*^c^* | 0.94 (0.81-1.08) | - |
| **Number of previous gestations**, median [IQR] | 3 [1.5-4] | 3 [2-4] | 0.864*^c^* | 1.05 (0.82-1.33) | - |
| **Literacy status (Literate)**, n (%) |  |  |  |  |  |
| **cannot read/write** | 10 (25.0) | 6 (15.4) | 0.288*^a^* | Reference | - |
| **can read/write** | 30 (75.0) | 33 (84.6) |  | 1.83 (0.59-5.66) | - |
| **Anemia (Hb<11 g/dL) at delivery**, n (%) |  |  |  |  |  |
| **not anemic** | 17 (42.5) | 20 (51.3) | 0.434*^a^* | Reference | - |
| **anemic** | 23 (57.5) | 19 (48.7) |  | 0.70 (0.29-1.71) | - |
| **HIV viral load at delivery**, median [IQR] | 0 [0-0] | 0 [0-0] | 0.039*^c^* | 0.9941 (0.9853-0.9852) | 0.9942 (.9846-1.0039) |
| **CD4 cells count at delivery**, median [IQR] | 543 [329-721] | 538 [386.5-917] | 0.491*^c^* | 1.0000 (0.9988-1.0012) | - |
| **Adherence to ART**, n (%) |  |  |  |  |  |
| **above 80%** | 35 (89.7) | 35 (97.2) |  | 4 (0.42-37.60) | 8.05 (0.52-125.65) |
| **below 80%** | 4 (10.2) | 1 (2.8) | 0.205*^d^* | Reference | Reference |
| **Days since last IPTp dose**, median [IQR] | 20 [14-31] | 26.5 [19-42] | 0.066*^c^* | 1.02 (1.00-1.05) | 1.03 (1.00-1.07) |

1. ***3TC cord blood (median conc. 222.4 ng/mL)***

| **3TC CORD BLOOD** | | | | | |
| --- | --- | --- | --- | --- | --- |
| **Characteristic** | **Below median**  **(n=33)** | **Above median**  **(n=33)** | ***P*-value** | **Univariate OR (95% CI)** | **Adjusted OR (95% CI)** |
| **Treatment (DHA-PPQ)**, n (%) |  |  |  |  |  |
| **Placebo** | 19 (57.6) | 14 (42.4) | 0.218*^a^* | Reference | Reference |
| **DHA-PPQ** | 14 (42.4) | 19 (57.6) |  | 1.84 (0.69-4.89) | 2.35 (0.82-6.74) |
| **Country (Gabon)**, n (%) |  |  |  |  |  |
| **Mozambique** | 27 (81.8) | 25 (75.7) | 0.547*^a^* | Reference | - |
| **Gabon** | 6 (18.2) | 8 (24.2) |  | 1.44 (0.49-4.73) | - |
| **Age**, mean (SD) | 28.76 (6.7) | 29.6 (5.9) | 0.615*^b^* | 1.02 (0.94-1.10) | - |
| **BMI**, mean (SD) | 25.11 (3.8) | 24. 7 (4.1) | 0.654*^b^* | 0.97 (0.86-1.10) | - |
| **MUAC**, mean (SD) | 27.93 (3.2) | 27.5 (3.6) | 0.565*^b^* | 0.96 (0.83-1.11) | - |
| **Gestational age recruitment**, mean (SD) | 19.33 (4.4) | 18.9 (4.5) | 0.681*^b^* | 0.98 (0.88-1.09) | - |
| **Gestational age delivery**, median [IQR] | 39 [38-41] | 40 [38-41] | 0.910*^c^* | 0.99 (0.81-1.21) | - |
| **Number of previous gestations**, mean (SD) | 2.64 (1.9) | 2.8 (1.9) | 0.744*^b^* | 1.05 (0.80-1.36) | - |
| **Literacy status (Literate)**, n (%) |  |  |  |  |  |
| **cannot read/write** | 7 (21.2) | 6 (18.2) | 0.757*^a^* | Reference | - |
| **can read/write** | 26 (78.8) | 27 (81.8) |  | 1.21 (0.36-4.09) | - |
| **Anemia (Hb<11 g/dL) at delivery**, n (%) |  |  |  |  |  |
| **not anemic** | 11 (33.3) | 19 (57.6) | 0.048*^a^* | Reference | Reference |
| **anemic** | 22 (57.6) | 14 (42.4) |  | 0.37 (0.14-1.00) | 0.40 (0.14-1.16) |
| **HIV viral load at delivery**, median [IQR] | 0 [0-0] | 0 [0-0] | 0.562*^c^* | 1.0001 (0.9998-1.0004) | - |
| **CD4 cells count at delivery**, median [IQR] | 505 [352-704] | 501.5 [339-819] | 0.782*^c^* | 0.9998 (0.9986-1.0011) | - |
| **Adherence to ART**, n (%) |  |  |  |  |  |
| **above 80%** | 29 (90.6) | 30 (96.8) | 0.613*^d^* | 3.10 (0.31-31.58) | - |
| **below 80%** | 3 (9.4) | 1 (3.2) |  |  | - |
| **Days since last IPTp dose**, median [IQR] | 21.5 [14.5-31] | 24 [13-42] | 0.380*^c^* | 1.02 (1.00-1.05) | 1.02 (0.99-1.05) |

Tables S2. a-d: Descriptive, univariate and multivariate logistic regression of Covariates on the outcome variable above and below study population medians.

Covariates were described above and below study population median as n (%), median [IQR] and mean (sd); p-values were calculated using *^a^*χ² test, *^b^*Student's t-test, *^c^*Wilcoxon rank-sum test, *^d^*Fisher’s exact tests.

Abbreviations: 3TC, lamivudine; DTG, dolutegravir; OR, odds ratio; CI, confidence interval; DHA-PPQ, dihydroartemisinin-piperaquine; EOP, end of pregnancy; MUAC, mid-upper arm circumference; BMI, body mass index; Hb, hemoglobin; ART, antiretroviral therapy; IPTp, Intermittent preventive treatment during pregnancy.

Odds ratios are adjusted for all variables included in the final multivariable model.
